# Hint-Based Image Colorization Based on Hierarchical Vision Transformer

**DOI:** 10.3390/s22197419

**Published:** 2022-09-29

**Authors:** Subin Lee, Yong Ju Jung

**Affiliations:** School of Computing, Gachon University, Seongnam 13120, Korea

**Keywords:** image colorization, vision transformer, attention map, deep learning

## Abstract

Hint-based image colorization is an image-to-image translation task that aims at creating a full-color image from an input luminance image when a small set of color values for some pixels are given as hints. Though traditional deep-learning-based methods have been proposed in the literature, they are based on convolution neural networks (CNNs) that have strong spatial locality due to the convolution operations. This often causes non-trivial visual artifacts in the colorization results, such as false color and color bleeding artifacts. To overcome this limitation, this study proposes a vision transformer-based colorization network. The proposed hint-based colorization network has a hierarchical vision transformer architecture in the form of an encoder-decoder structure based on transformer blocks. As the proposed method uses the transformer blocks that can learn rich long-range dependency, it can achieve visually plausible colorization results, even with a small number of color hints. Through the verification experiments, the results reveal that the proposed transformer model outperforms the conventional CNN-based models. In addition, we qualitatively analyze the effect of the long-range dependency of the transformer model on hint-based image colorization.

## 1. Introduction

As deep-learning technologies have evolved, there have been several advances in various fields of computer vision, with interesting advances in image colorization tasks based on convolutional neural networks (CNNs). Colorization is divided into (1) automatic colorization methods [1,2,3,4,5,6], which perform colorization on a fully black-and-white image and (2) hint-based colorization [7,8], in which the color values of some pixels are given as hints (i.e., color hints), and the remaining luminance pixels are colorized to create a full-color image.

The hint-based colorization method is used in various applications such as image editing and computational photography. A typical example is user-scribble-based colorization [7], which uses an image editing tool to perform colorization based on the color hints given in the form of scribbles by the user. Compared with fully automatic colorization, it has the advantage that the user can determine the image colorization direction and know how the image changes as the hints change. Another application of the hint-based colorization is the color-plus-monochrome dual camera [9], which consists of a pair of color and monochrome cameras. In particular, it is used to create better-quality images by colorizing the luminance pixels captured from a monochrome camera with the chrominance information captured from a color camera. This method allows users to capture image structures well in low-light shooting conditions. The hint-based colorization method is also used in applications to capture a high-quality full-color image using sparse color pixels from the sparse color sensor [10].

Some previous studies [7,8] have proposed user-interactive colorization methods when color hint pixels or a global color histogram are given as hints. Among them, the most notable study is a user-guided colorization method proposed by Zhang et al. [7]. This method takes the user’s color pixel hints as input and performs the hint-based colorization using a CNN model with an encoder-decoder structure.

However, in the case of CNN-based colorization, colorization is performed in a direction where the local characteristics are strong based on a method that applies convolution to sparse hint pixels and propagates the color information gradually to the surroundings. Hence, if some regions have insufficient hints or several objects are the same or similar in one image, but only some of them have hints, there is a limitation in that the colorization is performed incorrectly and thus the colorized results often suffer from visual artifacts such as color bleeding and false colors [9]. This problem occurs because color hints are propagated only to the local region and not to similar object regions that exist far away in an image.

Solving this problem requires the long-range dependency of the transformer model, which has recently gained attention. The Vision Transformer (ViT) [11] model has reconstructed the transformer [12] used in natural language processing to be used in images. Recent studies on ViT have reported that transformer-based models using the long-range dependency in image classification tasks may be more effective than CNN-based models. Subsequently, several methods that outperform CNN-based methods have been studied using the advantages of ViT in various computer-vision tasks [13,14,15]. Recently, the research direction of colorization has focused on automatic methods rather than hint-based methods. Consequently, studies have been conducted on transformers for automatic colorization, but not on transformer models for the hint-based colorization.

In this paper, we propose a hint-based colorization transformer network (HCoTnet). The proposed network has a hierarchical vision transformer architecture that outputs full-color channels in the CIELAB color space from the input luminance image and sparse color hint pixels. The proposed hierarchical vision transformer network is based on the encoder-decoder architecture, which has been often used in CNN-based models for image-to-image translation tasks, and each block of the encoder-decoder is constructed as transformer layers.

Our contributions are as follows:

- We propose a hint-based colorization transformer network HCoTnet, which can propagate the input color hint better to the entire image region through the long-range dependency of the transformer.
- As a network of the hierarchical vision transformer architecture with an encoder-decoder structure, the proposed HCoTnet shows good performance in hint-based colorization tasks. Through the verification experiments using the ImageNet dataset [16], we verify that the transformer-based method outperforms conventional CNN-based methods in both qualitative and quantitative measures. In addition, we qualitatively analyze the effect of the long-range dependency of the transformer model on hint-based image colorization.

The remainder of this paper is structured as follows. Section 2 introduces related studies. Section 3 describes the architecture and each component module of the proposed HCoTnet. Section 4 discusses the results of experiments comparing the proposed method and conventional methods. Finally, Section 5 presents the conclusion.

## 2. Related Work

### 2.1. Hint-Based Image Colorization Using CNN

Zhang et al. [3,7] proposed a user-guided image colorization method based on a CNN network. A user can guide the colorization task by providing local or global color hints. As local hints, the user can assign scribble-based color hints to certain pixels or areas of the grayscale image. Then, the proposed CNN model propagates the color from the scribble across the image. Also, the user can provide global statistics information (e.g., color histogram) and the model uses it as a global hint in the coloriziation task. The network structure of Zhang’s model is a Unet [17] that consists of an encoder part, bottleneck layers, and a decoder part. The user’s local or global hint is inserted in the encoder or bottleneck layers of the network. To propagate the hint over a wider region in the image, the dilated convolution [18] is used at the bottleneck layer.

In addition, some methods have been proposed for the colorization of line art or sketch images [8] and example-based colorization [19]. Ci et al. [8] proposed a conditional GAN-based colorization model that uses a grayscale line art image and user’s color strokes as inputs. The line art or sketch-based methods suffer from the lack of semantic information on the grayscale input and hence the results often show inaccurate and irregular shading artifacts. To overcome this, they proposed a conditional GAN-based approach. Also, Xiao et al. [19] proposed an example-based colorization method that uses another reference color image as a global hint. Given a grayscale image and a reference color image as inputs, the missing color is predicted by analyzing the color distribution of the reference image. To this end, they defined the image colorization as a multinomial classification problem and proposed a CNN-based model with a hierarchical pyramid structure.

Although the convolution-based methods show promising results in the colorization task, the strong locality characteristics of the convolution operation can cause severe visual artifacts such as color bleeding artifacts [9]. That is, some color hints given for an object can be sometimes spilled over its neighbouring objects due to the strong local dependency between the neighboring objects.

### 2.2. Vision Transformers

ViT [11] was the first model to apply the transformer used in the field of natural language processing to the computer vision domain. For the image classification task, the input image was divided into patches of size 16×16, and considering each one as a single word token, the model was constructed based on a transformer encoder comprising layer normalization (LN), multi-head attention (MHA), and multi-layer perceptron (MLP). In image classification, this model outperformed conventional CNN-based models and also showed good performance in other vision tasks, such as instance segmentation [20,21,22,23] and object detection [24,25,26].

In recent years, several advanced ViT methods have been proposed [13,14,15,27,28]. Liang et al. [14] have proposed a hybrid method that combined the transformer with CNN. This method is an attempt to restore the image by combining the transformer with the CNN encoder–decoder backbone. This method uses convolution blocks in the feature extraction and image reconstruction, and the bottleneck layer for the deep feature extraction consists of Swin transformer blocks [13]. Notably, in three image restoration tasks (super-resolution, denoising, and jpeg compression artifact reduction), this method outperformed CNN-based models, showing that the transformer-based architecture can be effectively used in the image restoration tasks as well.

Furthermore, TransGAN [15] has been proposed to check whether the transformer operates properly in complex and difficult image generation tasks based on a generative adversarial network (GAN) [29]. A GAN model was constructed by constructing a generator and a discriminator using the transformer, and it demonstrated high-quality image generation. It was confirmed that the transformer operates properly in image generation tasks, which require spatial consistency for the structure, color, and texture of the image.

In addition, a recent study [30] proposed a transformer-based method for the automatic image colorization task. The Coltran [30] has been proposed based on the Axial transformer [31]. This method uses the auxiliary parallel technique and the conditional transformer layer based on the row and column self-attention [31]. Specifically, the input grayscale is downsampled to a low-resolution image (e.g., 64×64). Then this coarse image is processed through the color upsampler and spatial upsampler to output a higher-resolution color image (e.g., 256×256). The results in the human evaluation showed that the transformer-based automatic colorization method outperforms the existing CNN method [32]. Based on the results, they concluded that the proposed method can generate visually plausible colors that cannot be distinguished from those of the ground-truth images by the human observers. However, their method is only valid for the automatic colorization task and there have been none thus far on the hint-based colorization task based on the vision transformer. In this paper, we construct the proposed network based on ViT [11], the basic transformer model, to show that the transformer can outperform conventional CNN models in the hint-based colorization task.

## 3. Method

**Figure 1:**
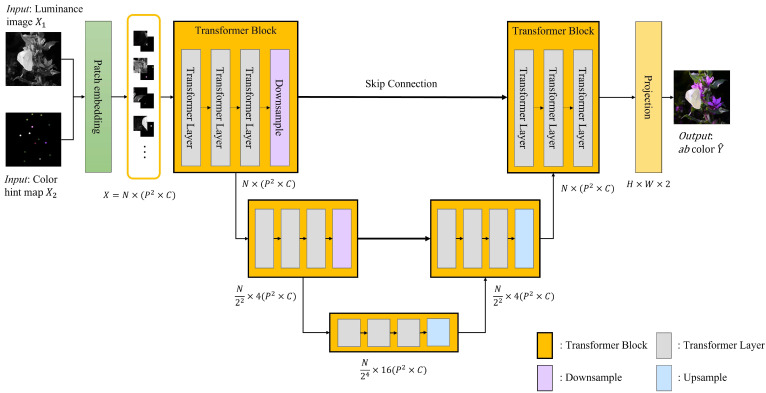
Overall architecture of the proposed hint-based colorization transformer network (HCoTnet).

As shown in Figure 1, the proposed HCoTnet is divided into three main parts: first, a patch embedding (tokenization) module for using the input data (i.e., luminance image and color hint map) in the transformer; second, the Unet-like encoder and decoder modules consisting of transformer blocks [17]; finally, a projection module for outputting the result by restoring and projecting the embedded features onto the ab dimension of the CIELAB color space. The luminance image and color hint map are the inputs of the network, and through the HCoT network, the colorization result is output with the ab color channels.

### 3.1. Patch Embedding (Tokenization)

As with previous studies [1,3,4,5,7,9], it is common to perform image colorization tasks in the CIELAB color space rather than in the RGB color space. The CIELAB color space is used due to its perceptually uniform characteristics and its similarity to the human visual system [33,34,35]. In addition, note that the RGB color space is an correlated color space between luminance and chrominance. In contrast, the CIELAB color space consists of two decorrelated channels between luminance and chrominance. The image colorization task aims at colorize the missing color information based on the given luminance information. Hence, using the CIELAB color space fits well with the colorization task by simplifying it to the prediction of only ab color channels using the input luminance data. Due to these reasons, the CIELAB has been widely used in many image synthesis tasks such as image inpainting [36] and image colorization [1,3,4,5,7,9]. Therefore, before the patch embedding, the input luminance image and RGB values of color hints are transformed into the CIELAB color space. The luminance image is constructed with the *L* channel, and the color hint is constructed with ab channels, thereby constructing a single Lab image.

With the constructed image, the patch embedding is performed in a manner similar to that of ViT [11]. The input $I\in R^{H\times W\times 3}$ (i.e., one *L* channel and two ab color channels for color hint) expands to H×W×4 channels through a convolution. Here, *H* denotes the image height, and *W* denotes the width. The input is then sliced into patches of size 4×4, and each patch has $4^{2}\times 4=64$ channels. The sliced patches are then transformed in a 1D sequence of size $N\times P^{2}\times C$, and finally, embedded patches *E* are constructed. Here, *N* is the total number of patches (N=H/4×W/4), *P* is the patch size = 4, and *C* is the channel size = 4. In our implementation, H=256, W=256, and $N\times (P^{2}\times C)=4096\times 64$. As class tokens are not suitable for the colorization task, unlike in ViT, they are not added, and convolutional positional embedding (CPE) [37,38,39] is used for the positional embedding. The CPE is used to replace the positional embedding in the ViT. As the CPE uses a small convolution, it can model position information effectively without affecting the total computational amount of the model. Then, the transformed patch token passes through the transformer layers, generating a feature representation.

### 3.2. Transformer Layer

**Figure 2:**
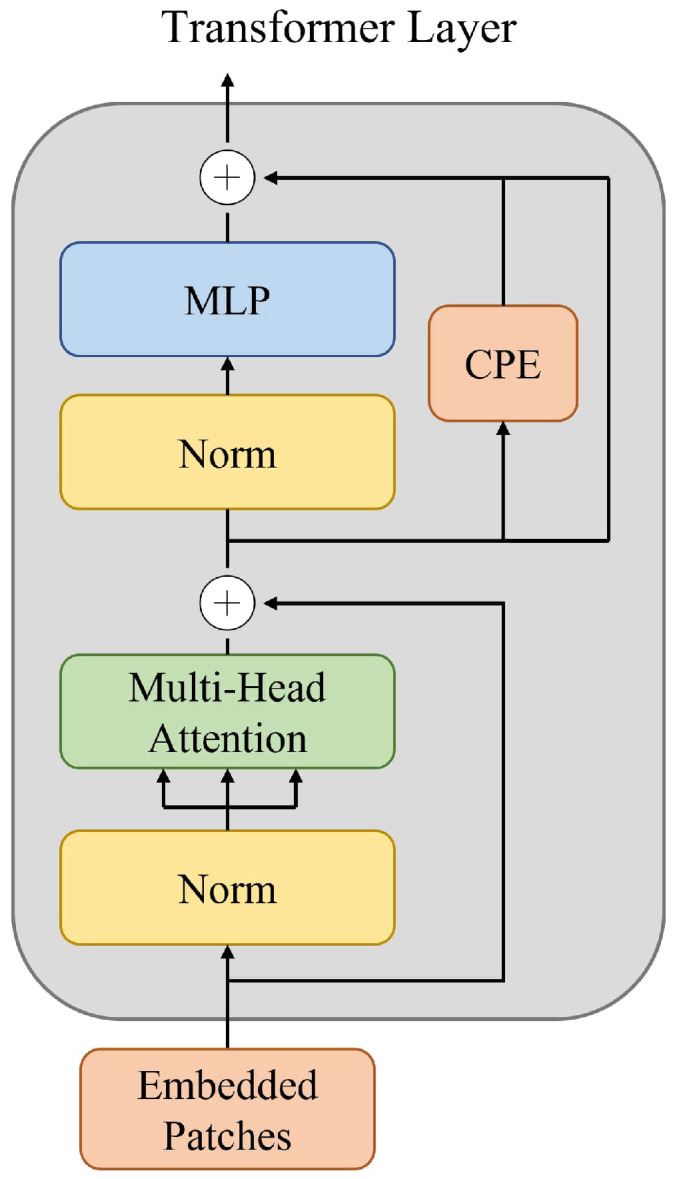
Structure of a transformer layer [11].

Figure 2 shows the transformer layer used in the proposed HCoTnet. The embedded patches pass sequentially through the transformer layer consisting of the LN, MHA, MLP, and CPE [11]. The operation in a transformer layer for the input $X_{l-1}$ from the previous transformer layer is defined as follows:

(1) $${X^{\prime }}_{l}=MHA\left(LN\left(X_{l-1}\right)\right)+X_{l-1},$$

(2) $$X_{l}=MLP\left(LN\left(X_{l}^{\prime }\right)\right)+CPE\left({X^{\prime }}_{l}\right)+{X^{\prime }}_{l},$$

where $X_{l}$ is the output of the *l*-th transformer layer.

As with the conventional ViT [11], the self-attention [12] in the MHA module is defined as follows:

(3) $$Attention\left(Q,K,V\right)=Softmax\left(\frac{{\mathrm{QK}}^{T}}{\sqrt{D}}\right)V,$$

where Q, K, and V represent the query, key, and value matrix, respectively. The correlation between each patch token is modeled through the QKV-attention [11]. As the correlation is modeled, the color hint features are delivered to similar embedded patches. In this way, color hint features can be smoothly propagated even between the patches away in the image.

The MLP consists of a hidden layer, which expands to four times the input dimension, and a Gaussian error linear unit (GELU) activation function. In CPE [37], Embedded patches are transformed from the embedded dimension (N×D) to the image dimension (D×H×W), and the group convolution [40] is performed with D groups, after which the patches are restored to the embedded dimension.

As shown in Figure 1, three transformer layers are connected sequentially in a transformer block. Based on the transformer blocks, the Unet [17] structure is constructed by downsampling or upsampling the feature map obtained after passing through all the transformer layers.

### 3.3. Encoder

In the encoder, semantic contexts are found gradually in the image, and the color hint is propagated between the patches that have similar contexts. As shown in Figure 1, the proposed model performs the spatial downsampling twice with two transformer blocks in the encoder, and after passing through the bottleneck transformer block, the decoder is connected.

Specifically, in the encoder process, the feature $X^{\left(i\right)}$ is obtained from the input $X^{\left(i-1\right)}$ after passing through the *i*-th transformer block constructed with three transformer blocks.

(4) $$X^{\left(i\right)}=f\left(X^{\left(i-1\right)}\right),where\;i=1,2.$$

Here, f() represents a transformer block, and *i* denotes the block number. Note that, in the first transformer block, the input $X^{\left(0\right)}$ represents the embedded patches E as constructed in Section 3.1. In our implementation, the output of the first block has a size of $X^{\left(1\right)}\in R^{4096\times 64}$. The features obtained this way are transformed from the embedded dimension to the image dimension. That is, in our implementation, the dimension of the embedded feature $X^{\left(1\right)}\in R^{4096\times 64}$ of the first block is converted into the image dimension $X^{\left(1\right)}\in R^{64\times 64\times 64}$ Then, the sizes of *H* and *W* are reduced to 1/2, and a spatial downsampling is performed through an operation of 2 × 2 convolution with stride = 2, which expands the channels by a factor of four.

(5) $${\hat{X}}^{\left(i\right)}=Downsample\left(X^{\left(i\right)}\right).$$

Note that, in the first block, the downsampled feature is ${\hat{X}}^{\left(i\right)}\in R^{256\times 32\times 32}$. Subsequently, it is restored to the embedded dimension (i.e., ${\hat{X}}^{\left(i\right)}\in R^{1024\times 256}$).

The above process is performed repeatedly with the next (i+1)-th transformer block to extract the feature. As the final step of the encoder, a high-level feature is extracted through the bottleneck layer consisting of a single transformer block.

**Figure 3:**
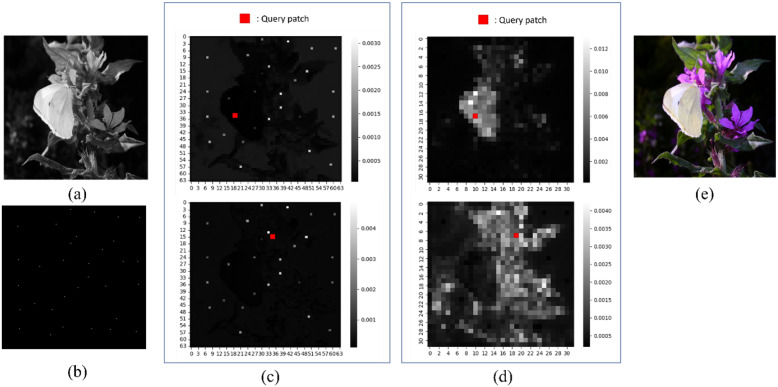
Visualization of the attention map in the transformer layer. (**a**) Input luminance. (**b**) Input color hint mask. (**c**) Self-attention map in the first transformer block of the encoder. (**d**) Self-attention map in the second transformer block of the encoder. (**e**) Ground-truth color image. Note that the attention maps in (**c**,**d**) are produced with each query patch, respectively.

Figure 3 shows an example of the self-attention maps extracted in the encoder stage. Note that these attention maps are extracted from the multi-head attention of the second transformer layer in each transformer block. As shown in Figure 3c, the attention map of the first transformer block in the encoder looks similar to the given hint mask (Figure 3b). This is because the propagation of hints has not yet been sufficiently accomplished in the first block of the encoder. Hence, high attention is assigned to the position of the hints rather than the image structures. Notably, the attention map shown in the top of Figure 3d is extracted from the second transformer block in the encoder, which is produced with a query patch of the butterfly’s wing. It can be seen that the attention map is composed of high attention according to the overall shape of the butterfly, and color hint information is transmitted smoothly from the inside. Also, the bottom image of Figure 3d shows the self-attention map for a query patch of the plant. It has high attention values in the plant areas except for the butterfly and background. This observation indicates that the transformer’s encoder can pay attention to similar objects in an image and hence correctly propagate the color hints even for long-distance but similar objects (We will further discuss this in Section 4.4).

### 3.4. Decoder

In the decoder, the color features that were globally propagated in the encoder are expanded through gradual upsampling. As in the encoding process, let ${\hat{Y}}^{\left(i\right)}$ be the feature after passing through the three transformer layers of the *i*-th transformer block of the decoder. After transforming the feature ${\hat{Y}}^{\left(i\right)}$ into the image dimension, an upsampling is performed.

(6) $$Y^{\left(i\right)}=Upsample\left({\hat{Y}}^{\left(i\right)}\right)$$

Here, the upsampling operation uses a pixel shuffle [41] with a factor of two to increase the height and width by 2 times and reduce the channels to 1/4. Note that this method uses a memory-friendly structure of TransGAN [15] to perform operations effectively while maintaining the total memory size of the embedding patches.

As with the conventional CNN-based Unet [17], as the structural information of the image may be partially lost in the downsampling process of the encoder, the encoder and decoder are connected through a long skip connection to restore the lost information. That is, the feature of the upper block obtained through one transformer block is as follows:

(7) $$Y^{\left(i-1\right)}=g\left(X^{\left(i-1\right)}+Y^{\left(i\right)}\right)$$

where f() is a transformer block, $X^{\left(i-1\right)}$ is the feature obtained from the (*i*-1)-th transformer block of the encoder, and $Y^{\left(i\right)}$ is the result obtained from the *i*-th transformer block of the decoder.

### 3.5. Projection

In the final stage, a projection is performed after passing through the final transformer block of the decoder. The projection stage consists of a process of restoring the feature from the embedded dimension to the image dimension and projecting it onto the ab dimension. As the patch slicing was performed with a size of 4×4 in the patch embedding stage, a single embedding token contains the pixel information of size 4×4. Therefore, a token is projected as 4×4×2 (i.e., for ab dimension). The embedded patches are transformed to the image dimension (i.e., $R^{\left((H/4)\times (W/4)\times 64\right)})$, and the projection is performed to a size of $R^{\left((H/4)\times (W/4)\times (4\times 4\times 2)\right)}$ through a 1×1 point-wise convolution. Finally, the patches are transformed to the input image size through the pixel shuffle [41] with a factor of four times to produce the final ab channel output $ab\in R^{\left(H\times W\times 2\right)}$. See the supplementary material for the details of the network architecture.

## 4. Experiments and Results

In this section, we compare conventional CNN-based colorization models [1,7,17] and the proposed transformer model quantitatively and analyze the results visually to examine how effectively the long-range dependency operates.

### 4.1. Experiment Setting

#### 4.1.1. Dataset

We trained and tested all the models using the ImageNet dataset [16], which is most widely used for classification tasks. Considering that, if the dataset belongs to some specific domain, the model can learn only the color information of the domain, we used ImageNet [16] to let the model learn colorization in various image categories (i.e., 1000 classes). Furthermore, as the transformer has low inductive bias, thus requiring a sizable dataset [11], we used ImageNet, which consists of 1.2 M images. For the main comparison experiments, the input size of an image was 256 × 256.

#### 4.1.2. Implementation Details

The model was implemented using PyTorch [42], and the training was performed using Nvidia RTX 3090 GPU with 24 GB memory. L1 loss was used for the training. The optimization was performed using Adam Optimizer [43] (beta1: 0.9, beta2: 0.999) and a learning rate of 0.00004.

#### 4.1.3. Comparison Methods

We used various conventional methods for comparison with the proposed method. First, the Unet model [17] consisted of an encoder and a decoder that perform downsampling and upsampling, respectively, with two scales. In the case of the Iizuka model [1], although it was originally an automatic colorization method using classification, it was modified to operate based on color hints by changing the grayscale input into an *L*-channel with ab hint channels on the input layer. In the case of the Zhang model [7], hint-based colorization was implemented, and the test was performed with the same training data and environments as the proposed method. In the case of ViT [11], we constructed the model by removing the MLP-head for classification after the transformer encoder and projecting the embedded patches onto the ab dimension.

#### 4.1.4. Evaluation Metrics

For quantitative evaluation metrics for the comparison, we used peak signal-to-noise ratio (PSNR) and structural index similarity (SSIM [44]), which are commonly used to evaluate the colorization performance. Moreover, we added the learned perceptual image patch similarity (LPIPS [45]) to check how much the colorization results are cognitively correct.

### 4.2. Visual Comparison

**Figure 4:**
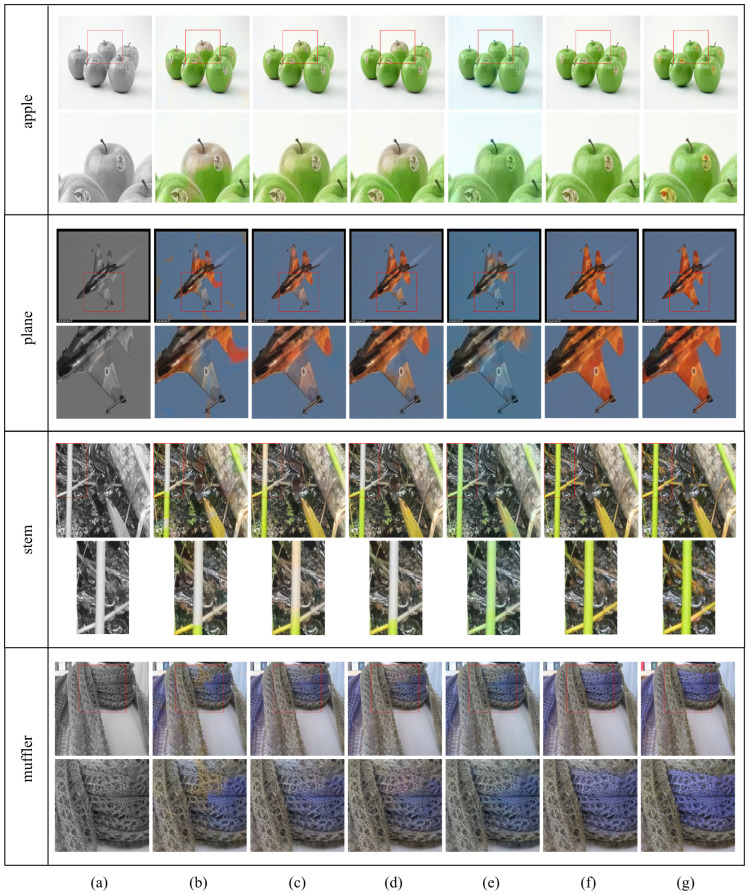
Visual comparison of hint-based colorization methods. (**a**) Input luminance image. (**b**) Results of Unet [17]. (**c**) Results of Iizuka [1]. (**d**) Results of Zhang [7]. (**e**) Results of ViT [11] (**f**) Results of the proposed HCoTnet. (**g**) Ground truth.

Figure 4 shows the colorization results given that the ratio of input hints to the total number of pixels in the image is 0.5%. That is, the color hints used in this experiment were obtained by randomly selecting 0.5% pixels from each ground-truth color image.

As shown in Figure 4b–d, the CNN-based models show incomplete colorization results in the red box area (see the magnified images of the red box area). In the “apple” images, the top part of the apple shows incorrect colorization results for the CNN-based models. This is because insufficient color hints were provided in that region. However, in the ViT and proposed transformer models, the same region was properly colorized as shown in Figure 4e,f.

In the “plane” images, the results of the CNN-based models show the color bleeding artifacts in the wing of the fighter plane. This region was colorized the same color as the background, as shown in Figure 4b–d. This is because there was no color hint around the boundary between the wing and the background, and the CNN-based models could not recognize the object’s region accurately. Similarly, in the result of the ViT, a wider region was colorized in blue, as shown in Figure 4e. Contrarily, the proposed model recognized the shape of the fighter plane accurately and used the long-range dependency in the other region where hints were insufficient, showing an appropriate colorization result.

In the “stem” images, the CNN-based models did not perform properly because of the lack of hints as observed in the apple image. However, in the case of the ViT and proposed models, the colorization was performed properly based on the long-range dependency using the hints at the lower part of the stem.

The last “muffler” image is the case where the muffler has two different colors and some of color hints are incorrectly provided by the user. The CNN-based models show the color bleeding artifacts occurred from the incorrect hints, as shown in Figure 4b–d. This can be because the CNN models incorrectly analyzed the muffler object’s region, and hence incorrect hints were spilled inside the same muffler object. Contrarily, the proposed model accurately colorized the muffler region so that it looks like the ground-truth image, even though the color hint was incomplete.

**Figure 5:**
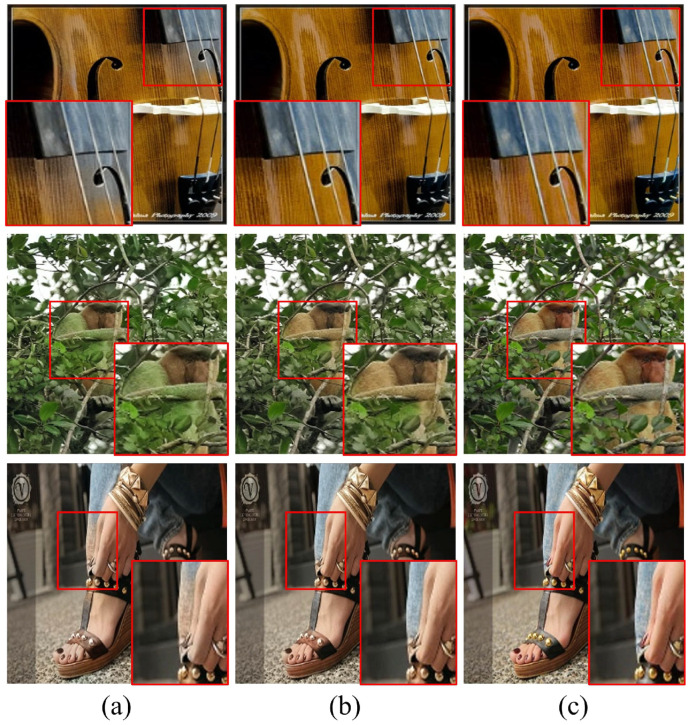
Comparison with respect to color bleeding artifacts. (**a**) Results of Zhang’s CNN model [7]. (**b**) Results of the proposed HCoTnet. (**c**) Ground truth.

Figure 5 shows the visual result for the color bleeding artifacts, which are important in colorization tasks. In the case of the CNN-based models, colorization was not achieved correctly, or the color bleeding occurred in some regions where hints were insufficient. In the case of the proposed model, even in regions where hints were insufficient, colorization was performed as much as possible using the hint information found slightly far away. Furthermore, the proposed model showed a tendency to naturally perform colorization even for objects that did not have hints, by using the color hint of a similar object. These results show that the proposed transformer-based colorization method has fewer color bleeding artifacts than the conventional CNN-based methods. See the supplementary material for more visual examples.

### 4.3. Quantitative Comparison

Table 1 shows the quantitative comparison results for the conventional CNN models (Unet [17], Iizuka [1], Zhang [7]), a ViT model [11], and the proposed model. The test results were obtained using the color hints made by randomly selecting 0.5% pixels from each ground-truth image of the test dataset.

As seen in Table 1, the proposed transformer-based model demonstrated the best performance, achieving a PSNR of 31.645 dB, a SSIM of 0.895, and a LPIPS of 0.043. Particularly, the proposed method showed a performance improvement of PSNR over 1 dB compared with all the CNN-based models (i.e., Unet [17], Iizuka [1], and Zhang [7]). The same tendency was observed in terms of the SSIM and LPIPS. As mentioned in the visual comparison, this performance improvement is attributed to that the proposed model generates fewer regions with the color bleeding and incomplete color artifacts than those of the CNN-based models. The ViT model [11] showed the worst performance in terms of all the metrics. Although it also uses the transformer’s long-range dependency for the regions where the hint is insufficient, it’s network architecture is originally designed for the image classification tasks, and hence it is not suitable for the image generation tasks such as colorization.

Table 2 shows the comparison results obtained by changing the color hint ratio given as an input. At every hint ratio, the proposed model achieved the best performance. Notably, as the hint ratio decreased, the difference from the CNN models increased. This indicates that the fewer the hints, the more useful the long-range dependency of the transformer is and the better the results are. However, the experimental results of ViT showed a significant decline in performance compared with that of the CNN-based models. In particular, when the hint ratio was 0.1%, colorization was not performed correctly at all. This also indicates that the basic architecture of ViT is not suitable for the image generation and synthesis tasks. Hence, a transformer of the encoder-decoder architecture is required, as in the proposed model.

In addition, we have measured the running time of the proposed model and compared with that of the CNN-based baseline model (i.e., Zhang’s model). The running time was calculated by averaging the running time for 100 images. It was measured using a Nvidia RTX 3090 with 24 GB. The running time of the proposed model was 27.28 ms and the Zhang’s model was 8.36 ms. The proposed transformer-based model showed a longer running time than that of the CNN-based model because the multi-head attention operation has a high computation complexity, as mentioned in literature [12,13,15]. Note that the running time of our model is similar to that of the existing transformer-based models for other image synthesis tasks (e.g., StyleSwin [46]: 27.94 ms, Uformer [47]: 27.19 ms, which are measured in the same computing environments).

### 4.4. Effect of Long-Range Dependency on Hint-Based Colorization

This section compares the conventional CNN models and the proposed transformer model to explain their effects on the hint-based colorization in terms of the long-range dependency.

**Figure 6:**
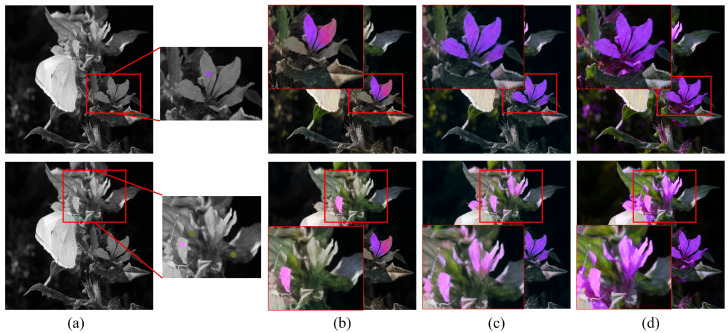
Comparison of hint-based colorization. (**a**) Input luminance and color hints. (**b**) Results of a CNN (Zhang’s method [7]). (**c**) Results of our transformer-based colorization. (**d**) Ground truth. Note that the transformer-based method outperforms the CNN-based method because it considers rich long-range dependencies. Color hints have been enlarged for visual representation.

Figure 6 shows an example demonstrating the importance of long-range dependency in hint-based colorization. The first row in the figure shows how colorization is performed when there is no color hint in an object. In the case of Zhang’s CNN-based model [7], the flowers in the region where there was no color hint were colorized using the green color hint in the surroundings. On the other hand, in the case of the proposed transformer-based model, the flowers were colorized in purple based on the color hint information of other flowers using the long-range dependency. The images in the second row show the results for the case where a hint is given in only one of the four petals. In the case of Zhang’s model [7], the petal with the hint and the petals on the right side within the region that could be covered by the receptive field were colorized correctly. But the rest were not colorized, showing incomplete colorization. In the case of the proposed transformer model, colorization was performed properly using the color information of the petal that had the hint. As such, long-range dependency is important in hint-based image colorization tasks [48].

**Figure 7:**
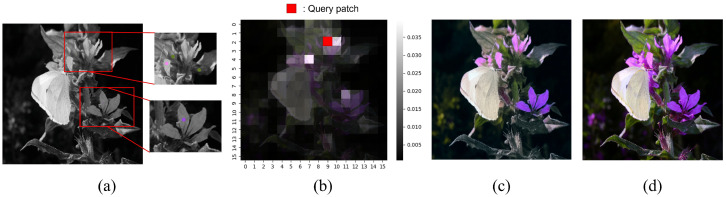
Visualization of the attention map in the bottleneck transformer layer. (**a**) Input (luminance and color hint). (**b**) Self-attention map in the bottleneck layer. (**c**) Visual result of HCoTnet. (**d**) Ground-truth color image.

Figure 7 shows a detailed analysis of this result. The input image (Figure 7a) contains the part where the color hint for the flower exists, which is the flower part with the butterfly and the flower leaf in the lower right of the image. Figure 7a shows two flower regions where the color hint exists. We can see that a few flower petals have color hints. Figure 7b shows the attention map associated with a query patch in a flower region. We can see that the petal located in the query patch has high attention for the other patch where the color hint exists. Note that the self-attention map is extracted from the bottleneck layer of the proposed HCoT model. Figure 7c shows the colorization result of the HCoT model. It can be seen from Figure 7c that most of the flower petals can be correctly colored by using the color hint given for other flower petals. This observation reveals the long-range dependency characteristics of the transformer model that can pay attention to the proper regions with color hints and similar objects.

**Figure 8:**
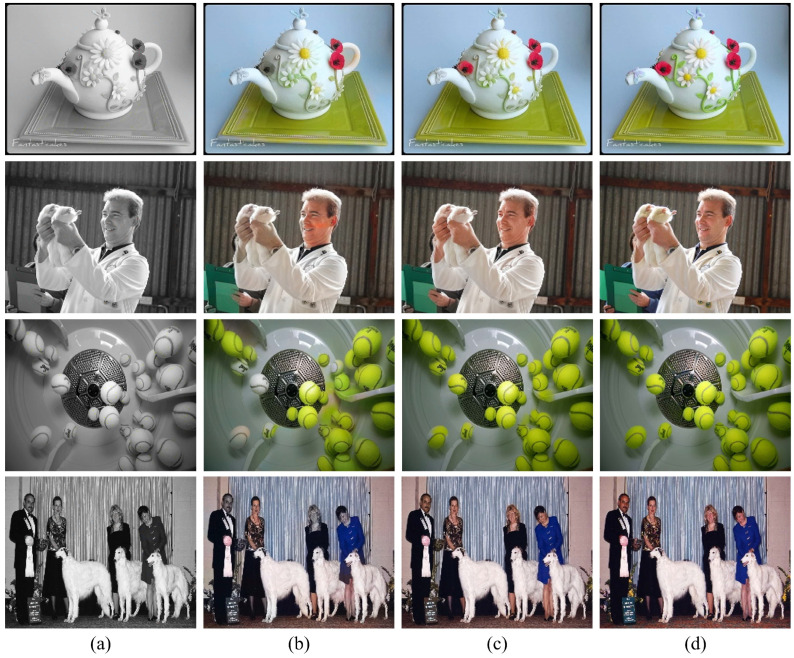
Visual comparison of the effect of long-range dependency on hint-based image colorization. (**a**) Input luminance and color hints. (**b**) Result of Zhang’s CNN model [7]. (**c**) Result of the proposed HCoTnet model. (**d**) Ground truth.

Furthermore, Figure 8 shows the effect of long-range dependency for the proposed HCoT model, compared with that of a CNN-based colorization model (i.e., Zhang’s method [7]). In the images in the first row, the Zhang model, a CNN-based method, did not perform colorization properly despite the presence of a color hint in the red flower decoration part in the left part of the pot. In contrast, the proposed model performed colorization correctly by additionally using the color hint of other flower decorations based on the long-range dependency. Furthermore, in the case of a small red flower decoration on the pot lid, the Zhang model could not colorize it because there was no hint, but the proposed model generated visually plausible results based on the color of other flower decorations.

In the images in the second row, the Zhang model did not colorize the left region of the boundary divided by the nose in the man’s face part. In contrast, the proposed model colorized it correctly. This indicates that the transformer model propagates hints in the same object smoothly. In the same aspect, there were multiple hint pixels in the man’s hand part, but the Zhang model did not colorize it with the correct skin color, whereas the proposed model colorized it accurately.

In the images in the last row, there was no hint in the face part of the woman dressed in blue on the right side of the image. Therefore, the Zhang model could not colorize the face part, but the transformer-based model used the long-range dependency to colorize it appropriately based on the color hint information in the face of another person. As in the case of the images in the second row, even though there were some hints in the face of the man on the leftmost side and the woman wearing a black dress, the Zhang model could not propagate them properly, being unable to perform colorization. However, the proposed model propagated them accurately and colorized everyone appropriately.

These results show that the long-range dependency of the transformer is used as an effective and appropriate method in hint-based colorization, producing better results.

### 4.5. Results on Higher Resolution Images

In this section, we provide the results of an additional experiment on much larger images. In this experiment, we have used a subset that consists of 7500 images randomly selected from the ImageNet testset and resized the image sizes as 512×512 and 1024×1024, respectively. To test our method for larger images without retraining the model, we divided an image into the subregions with a size of 256×256, run each region, and merged the results for the subregions into a final output. Table 3 shows the results of this experiment on larger images. As shown in the table, the performance of the proposed method outperforms the baseline CNN method (i.e., Zhang’s method [7]) in terms of all the metrics (PSNR, SSIM, and LPIPS).

## 5. Conclusions

In this study, we proposed a vision transformer network [11] with an encoder-decoder architecture for hint-based colorization. Through validation of the proposed model, we showed that the long-range dependency of the transformer can work effectively in hint-based colorization tasks. Even in regions where there were insufficient or no hints, the proposed model showed better results using color hints of similar objects in the image based on the long-range dependency. Furthermore, the experiments proved that the fewer hints, the greater the effect of the long-range dependency is. However, the use of transformers in the image synthesis field requires considerable computational complexities and computing resources. In the future, developing a method for reducing computational complexity and computing resources while maintaining the effect of long-range dependency will be an important challenge that will allow transformers to be used more smoothly in the image synthesis field.

## Figures and Tables

**Table 1:** Quantitative comparison results. Note that the results were obtained using 0.5% color hints.

Method	PSNR	SSIM	LPIPS
Unet [17]	31.746	0.885	0.049
lizuka [1]	31.636	0.883	0.057
Zhang [7]	31.973	0.888	0.051
ViT [11]	28.764	0.844	0.085
HCoTnet (ours)	32.645	0.895	0.043

**Table 2:** Experimental results by color hint ratio.

Method	Color Hint 1%			Color Hint 0.5%			Color Hint 0.1%		
	PSNR	SSIM	LPIPS	PSNR	SSIM	LPIPS	PSNR	SSIM	LPIPS
Zhang [7]	32.845	0.896	0.04	31.973	0.888	0.051	28.214	0.843	0.096
ViT [11]	31.085	0.876	0.07	28.764	0.844	0.085	10.222	0.334	0.474
HCoT (ours)	33.301	0.902	0.035	32.645	0.895	0.043	30.351	0.872	0.067

**Table 3:** Experimental results on higher resolution images.

Image Size	512 × 512			1024 × 1024		
Metric	PSNR	SSIM	LPIPS	PSNR	SSIM	LPIPS
Zhang [7]	33.234	0.901	0.065	35.352	0.920	0.075
HCoT (ours)	34.384	0.911	0.052	36.660	0.930	0.061

## Data Availability

Not applicable.

## Supplementary Materials

The following supporting information can be downloaded at: https://www.mdpi.com/article/10.3390/s22197419/s1, Section 1: Supplementary Details; Figure S1: Visual Results.
