# Supplementary material for: Hint-Based Image Colorization Based on Hierarchical Vision Transformer
Source: Sensors (Basel). 2022 Sep 29;22(19):7419. doi: 10.3390/s22197419 (PMC9570951; doi:10.3390/s22197419)
Supplement: Supplementary file 1 [file sensors-22-07419-s001.zip › sensors-1922128-SI.pdf]

Supplementary Materials: Hint-Based Image Colorization Based on Hierarchical Vision Transformer

Subin Lee and Yong Ju Jung *

1. Supplementary Details

This supplementary material is accompanied by the manuscript "Hint-based Image Colorization based on Hierarchical Vision Transformer" and contains the network architectures and additional visual results.

Table S1 shows the network architecture of the patch embedding block. Table S2 shows the structure of a transformer block used in the encoder and decoder. Table S3 shows the structure of a transformer layer used in each transformer block. Table S4 shows the network architecture of the encoder module that consists of three transformer blocks including a bottleneck layer. Table S5 shows the structure of the decoder module. Table S6 shows the structure of the projection block.

Figure S1 shows additional visual comparison results in the ImageNet dataset [1].

References

1. Deng, J.; Dong, W.; Socher, R.; Li, L.J.; Li, K.; Fei-Fei, L. Imagenet: A large-scale hierarchical image database. In Proceedings of the 2009 IEEE/CVF Conference on Computer Vision and Pattern Recognition. Ieee, 2009, pp. 248–255.
2. Dosovitskiy, A.; Beyer, L.; Kolesnikov, A.; Weissenborn, D.; Zhai, X.; Unterthiner, T.; Dehghani, M.; Minderer, M.; Heigold, G.; Gelly, S.; et al. An image is worth 16x16 words: Transformers for image recognition at scale. *arXiv preprint arXiv:2010.11929* **2020**.
3. Zhao, Y.; Wang, G.; Tang, C.; Luo, C.; Zeng, W.; Zha, Z.J. A battle of network structures: An empirical study of cnn, transformer, and mlp. *arXiv preprint arXiv:2108.13002* **2021**.
4. Shi, W.; Caballero, J.; Huszár, F.; Totz, J.; Aitken, A.P.; Bishop, R.; Rueckert, D.; Wang, Z. Real-time single image and video super-resolution using an efficient sub-pixel convolutional neural network. In Proceedings of the IEEE/CVF Conference on Computer Vision and Pattern Recognition, 2016, pp. 1874–1883.
5. Ronneberger, O.; Fischer, P.; Brox, T. U-net: Convolutional networks for biomedical image segmentation. In Proceedings of the International Conference on Medical Image Computing and Computer-Assisted Intervention. Springer, 2015, pp. 234–241.
6. Iizuka, S.; Simo-Serra, E.; Ishikawa, H. Let there be color! Joint end-to-end learning of global and local image priors for automatic image colorization with simultaneous classification. *ACM Transactions on Graphics (ToG)* **2016**, *35*, 1–11.
7. Zhang, R.; Zhu, J.Y.; Isola, P.; Geng, X.; Lin, A.S.; Yu, T.; Efros, A.A. Real-time user-guided image colorization with learned deep priors. *arXiv preprint arXiv:1705.02999* **2017**.

Table S1. Structure of the Patch Embedding (Tokenization) block

Branches	Input	Operation	Parameters	Output	Output Dimension
Input			Input CIELab Image (L channel, ab hint channel)		
conv	Input	Convolution	(kenrel_size=3 $\times$ 3, stride=1, padding=1, out_channels=4)	feature	$4 \times H \times W$
patch_embedding	feature	Convolution	(kenrel_size=4 $\times$ 4, stride=4, padding=0, out_channels=4 $\times$ 4$\times$in_channels)	embedded_feature	$C \times (H/4) \times (W/4)$
reshape	embedded_feature	-	-	embedded_patches	$N \times (P^2 \times C)$
Output			Embbed Patches $(N \times (P^2 \times C))$		

Table S2. Structure of a Transformer Block

Branches	Input	Operation	Parameters	Output	Output Dimension
Input			Embedded_patch / previous Transformer Block output		
trans_layer1	Input	Transformer Layer	Transformer Layer parameters	trans_layer_feature1	$N \times D$
trans_layer2	trans_layer1	Transformer Layer	Transformer Layer parameters	trans_layer_feature2	$N \times D$
trans_layer3	trans_layer2	Transformer Layer	Transformer Layer parameters	trans_layer_feature2	$N \times D$
Output			Transformer Block Output $(N \times D)$		

Table S3. Sturcuture of a Transformer Layer used in the Transformer Blocks

Branches	Input	Operation	Parameters	Output	Output Dimension
Input			Embedded patch / previous Transformer Layer output		
patch_attention	Input	Multi-head Attention [2]	(num_head=8, qkv_bias=True, attention_drop=0.0, projection_drop=0.0)	attention_feature	$N \times D$
layer_norm1	attention_feature	Layer Normalization	(eps=1e-6)	normalized_feature1	$N \times D$
residual_connection			Sum (Input, normalized_feature1)		
mlp	residual_connection	Multi-Layer Perceptron	(mlp_ratio=4.0, Activation=GELU, dropout=0.0)	mlp_feature	$N \times D$
layer_norm2	mlp_feature	Layer Normalization	(eps=1e-6)	normalized_feature2	$N \times D$
reshape	residual_connection	-	-	reshape_feature	$C \times H \times W$
cpe [3]	reshape_feature	Group Convolution	(kernel_size=3 $\times$ 3, stride=1, padding=1, group=C)	cpe_feature	$C \times H \times W$
reshape	cpe_feature	-	-	reshape_cpe_feature	$N \times D$
residual connection			Sum (residual_connection, normalized_feature2, reshape_cpe_feature)		
Output			Transformer Layer Output $(N \times D)$		

Table S4. Structure of the Encoder

Branches	Input	Operation	Parameters	Output	Output Dimension
Input			Embedded_patch		
encoder1	Input	Transformer Block	Transformer Block parameters	encoder_feature1	$N \times (P^2 \times C)$
downsampling1	encoder_feature1	Convolution	(kernel_size=2 $\times$ 2, stride=2, padding=0, out_channels=in_channels$\times$4)	down_feature1	$(N/2^2) \times 4(P^2 \times C)$
encoder2	down_feature1	Transformer Block	Transformer Block parameters	encoder_feature2	$(N/2^2) \times 4(P^2 \times C)$
downsampling2	encoder_feature2	Convolution	(kernel_size=2 $\times$ 2, stride=2, padding=0, out_channels=in_channels$\times$4)	down_feature2	$(N/2^4) \times 16(P^2 \times C)$
bottleneck	down_feature2	Transformer Block	Transformer Block parameters	encoder_feature3	$(N/2^4) \times 16(P^2 \times C)$
Output			Encoder Output $(N/2^4) \times 16(P^2 \times C)$		

Table S5. Structure of the Decoder

Branches	Input	Operation	Parameters	Output	Output Dimension
Input			Encoder Output		
upsampling1	Input	Pixel Shuffle [4]	(upscale_factor=2)	up_feature1	$(N/2^2) \times 4(P^2 \times C)$
skip_connection			Sum (up_feature1, encoder_feature2)		
decoder1	skip_connection	Transformer Block	Transformer Block parameters	decoder_feature1	$(N/2^2) \times 4(P^2 \times C)$
upsampling2	decoder_feature1	Pixel Shuffle [4]	(upscale_factor=2)	up_feature2	$N \times (P^2 \times C)$
skip_connection			Sum (up_feature2, encoder_feature1)		
decoder2	skip_connection	Transformer Block	Transformer Block parameters	decoder_feature2	$N \times (P^2 \times C)$
Output			Decoder Output $N \times (P^2 \times C)$		

Table S6. Structure of the Projection block

Branches	Input	Operation	Parameters	Output	Output Dimension
Input			Decoder output		
reshape	Input	-	-	reshape_feature	$C \times (H/4) \times (W/4)$
projection	Input	Convolution	(kernel_size=1$\times$1, stride=1, padding=0, activation=hyperbolic tangent)	projection_feature	$32 \times (H/4) \times (W/4)$
expand	projection_feature	Pixel Shuffle	(upscale_factor=4)	ab_output	$2 \times H \times W$
Output			Model Output $(2 \times H \times W)$		

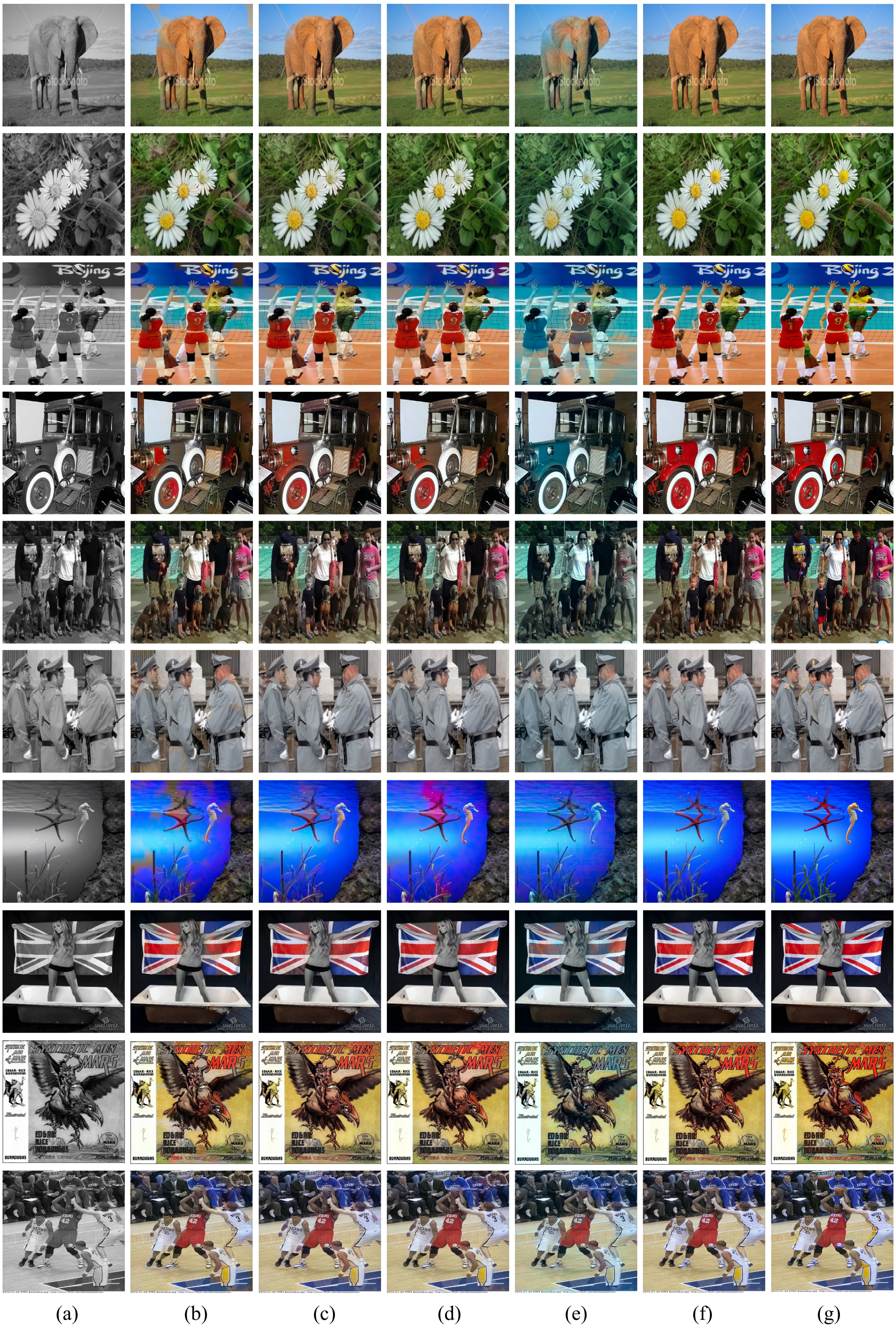

Figure S1. Additional visual comparison of hint-based colorization methods. (a) Input luminance image. (b) Results of Unet [5]. (c) Results of Iizuka [6]. (d) Results of Zhang [7]. (e) Results of ViT [2] (f) Results of the proposed HCoTnet. (g) Ground truth
